# Embodiment of intersubjective time: relational dynamics as attractors in the temporal coordination of interpersonal behaviors and experiences

**DOI:** 10.3389/fpsyg.2014.01180

**Published:** 2014-10-31

**Authors:** Julien Laroche, Anna Maria Berardi, Eric Brangier

**Affiliations:** ^1^Akoustic Arts R&D LaboratoryParis, France; ^2^PErSEUs, Université de LorraineMetz, France

**Keywords:** coordination, intersubjectivity, dynamical systems, embodiment, attractor, enaction, phenomenology, complexity

## Abstract

This paper addresses the issue of “being together,” and more specifically the issue of “being together in time.” We provide with an integrative framework that is inspired by phenomenology, the enactive approach and dynamical systems theories. To do so, we first define embodiment as a living and lived phenomenon that emerges from agent-world coupling. We then show that embodiment is essentially dynamical and therefore we describe experiential, behavioral and brain dynamics. Both lived temporality and the temporality of the living appear to be complex, multiscale phenomena. Next we discuss embodied dynamics in the context of interpersonal interactions, and briefly review the empirical literature on between-persons temporal coordination. Overall, we propose that being together in time emerges from the relational dynamics of embodied interactions and their flexible co-regulation.

## INTRODUCTION

How can we “share a moment” and experience this sharing? How can we share some time, even if it is immaterial? How can we share the intimacy of a moment despite the distance that usually separates our bodies? How can we feel being together if this means more than being in the same place or doing the same thing? For time is often taken for granted as an objective and physical dimension of reality, the issue of sharing its lived experience has not been much addressed by cognitive sciences. The aim of this paper is to provide a theoretical, phenomenological, and empirically grounded framework that addresses this issue.

To do this, we rely on the complementary approaches of phenomenology, enaction and dynamical systems (Froese and Gallagher, 2012). We take embodiment, temporality and interactivity seriously, and it is on the basis of these three inter-related dimensions that we construct our proposition. More precisely, since being a body is necessary for us to live (and therefore share) experiences, we first define what we mean by embodiment. In a second part, we address the issue of time as embodied, that is, the issue of how time is experienced and what kind of temporality underlies our embodiment. We then address the issue of embodiment in the context of intersubjectivity, and more specifically the issue of the embodiment of a properly intersubjective time. We finally discuss our overall proposition.

## WHAT IT IS TO BE EMBODIED

The notion of embodiment refers to numerous meanings (e.g., Wilson, 2002). In this section, we specify our understanding of what it is to be embodied through the lens of the enactive approach (Varela et al., 1991; Di Paolo, 2005; Thompson, 2007; McGann et al., 2013; Di Paolo and Thompson, 2014) and its phenomenological background (Husserl, 1913, 1931, 1952; Merleau-Ponty, 1942, 1945). According to the enactive approach, mind is both a *living* (observable, biological) and a *lived* (experienced) phenomenon that emerges from agent∼world coupling ^1^. Since living and lived aspects are concretely intertwined (Thompson and Varela, 2001), they can only be distinguished from an observer’s point of view. By abstraction, we discuss them successively; their entanglement will then become explicit.

### EMBODIMENT AS A LIVING PHENOMENON

At the roots of the enactive approach, living has been defined as the self-production and self-maintenance of its own organization, where “organization” means “the relations that exist among component processes of a system” (Varela et al., 1974; Varela, 1979; Maturana and Varela, 1987). It is thus a network whose operations are closed (i.e., each process has causes in and effects on other processes of the system). This interdependency enables the self-organized emergence of a coherent living unit. Emergence designates two complementary processes: the “local to global” formation of a new system (or pattern) out of the interactions between coupled components (i.e., out of the reciprocal effects they have on each other), and the “global to local” constraints that the newly formed system exerts on its components and the organization of their relations (Thompson and Varela, 2001). By producing itself, the living system actively “affirms” its own identity (it specifies what it is), and thereby defines its own intrinsic laws or norms of self-maintenance. In a word, the living system is auto-nomous (Varela, 1979; Di Paolo, 2005; Barandiaran and Egbert, 2014).

However, the autonomy of the living system is bounded by the domain of its viable relations with the environment. The boundaries of the living are therefore relational (rather than merely “skin-bounded”). Further, by generating itself, the living unit distinguishes itself from what it is not and thereby defines what the environment is from its own point of view (i.e., what counts as a significant environment and which value their relation has for the maintenance of its autonomous existence). The living unit thus constitutes an autonomous perspective on its own relations: interactions with the environment are asymmetrically anchored in its own, self-constituted perspective (Barandiaran et al., 2009). The phenomenological domain of the living is thus autonomous∼relational, which means both that the living system’s interactions are autonomous and that the autonomy of that system is realized interactively.

When a living system has the ability to regulate its coupled relations with the environment (as a function of the values that emerge from its own norms), we speak of an embodied agent whose interactive autonomy is adaptive (Di Paolo, 2005; Barandiaran et al., 2009): something has to be done to bring forth a “difference that makes a difference” (to quote the slogan of Bateson, 1972), preferably in the right direction (i.e., in accordance with its self-constituted norms). Cognition is thus broadly defined as a sense-making activity (Weber and Varela, 2002; Di Paolo, 2005): it consists in the enactment of a world of significance and values through autonomous interactions with the environment. In short, cognitive experiences are enacted from an autonomous perspective that is intrinsically relational.

### EMBODIMENT AS A LIVED PHENOMENON

In contrast of the above definition, classical accounts attribute cognition the role of mentally and internally representing information coming from the external world (Varela, 1988). Agent and world, organism and environment, subject and object or inner and outer are thus defined as being *a priori* external to each other. From a phenomenological point of view however, and as shown throughout this text, these boundaries are not given. These opposite poles only exist in the dynamics of their irreducible relations. Indeed, in lived experience, (cognizing) subject and (cognized) object are irreducible (Husserl, 1952), just like we can not distinguish the look from the thing that is seen. The detached, reflective stance is thus not our primary way of being in the world. Rather, our connection with the world is primarily corporeal and pre-reflective (Merleau-Ponty, 1945), as discussed below.

Appearance of our lived world obviously depends on our sensory structures, but motility directly affects how these sensory structures are perturbated by the environnement: what we do changes what can be sensed. The lived world is thus imprinted by our sensorimotor embodiment and is constituted in the context of our ongoing activity (McGann, 2010; McGann et al., 2013). Sensorimotor coupling allows for coherence of both the autonomous agent (its embodied experiences and its underlying internal dynamics) and his relations with the world. This is reflected in his own active and sensitive way of inhabiting the world he enacts (Buhrmann et al., 2013).

To discuss our pre-reflective connection with the world, we refer to the phenomenological distinction between the living body and the lived body. The living body refers to the image one can have of a body (or one’s own body), observed and thematized as an object of perception. The lived body is the pragmatic, unthematized (hence pre-reflective) background of experience, it is what our body-in-the-world affords us to sense and do (Lenay, 2010). This bodily self-consciousness is necessary for our experiences to be and feel “for” us, (Thompson, 2007). It is transparent to us: it is the pre-reflective background of our perspective, the point from which we see, do and live. In turn, affordances of the lived body are constantly reshaped by the ongoing activity of the living body: we enact the pre-reflective background of our perspective. Living and lived body thus co-constitute each other, and this is what defines embodiment (Thompson and Varela, 2001). It provides us with an autonomous perspective on our relations with the world (the phenomenal world that we enact and inhabit). Because both the co-constitution of lived and living body and the intertwinement of autonomy and relations are dynamical, we now turn our attention to the temporality of embodiment.

## THE EMBODIED MIND IN TIME

To address the issue of embodiment and temporality, we first present a phenomenological account of the time as lived, or time consciousness. Then, we address the issue of the temporality of the living. The co-constitution of lived temporality and temporality of the living will then be explicited.

### TIME CONSCIOUSNESS

Time consciousness is directed toward both the “outer” objects or events that have a temporal extension, and the “inner” experience of duration itself (i.e., the feeling of living enduring experiences with a temporal envelope; Thompson, 2007). This outer∼inner separation is only an abstract description from an external observer’s point of view: these aspects are irreducible in concretely lived experience. Indeed, we do not have an experience of the temporal extension of objects or events on the one side, and a sensation of our own enduring temporal experiences on the other: these aspects manifest themselves as a whole in a unified way (Thompson, 2007).

Husserl (1928) and its commentators (e.g., Merleau-Ponty, 1945; Varela, 1999a; Zahavi, 2003; Thompson, 2007; Gallagher and Zahavi, 2014) proposed a descriptive structure that accounts for both outer and inner time consciousness as well as their non-separateness. This structure consists in three inter-related component processes: primal impression, retention and protention. Primal impression designates the openness to the current “now-phase” of an object. This “now” is never lived in isolation of its temporal horizons, for there would be no time-extended perception (duration, succession or change) if present was lived as a succession of inarticulate moments (Varela, 1999a; Gallagher and Zahavi, 2014). Primal impression thus only exists in the networked conjunction with retention and protention. Retention is the subjective holding of the just-elapsed phase of the object or event that is receding into the past. Protention intends the phase of the object or event that is just about to occur: it is the temporal horizon formed by the (implicit) anticipation of the unfolding of experience.

These component processes do not behave “additively” (Gallagher and Zahavi, 2014): in the fullness of concrete experiences, they can’t be separated so as to manifest themselves as “retention + primal impression + protention” (i.e., they do not provide with diachronic feelings such as distinctly articulated past, present and future). Indeed, primal impression is qualified by both retention and protention: “now” would be different in the context of another retention and implicit anticipation. In turn, primal impression shapes what temporal horizon might be anticipated, and (re)shapes the way its retentional background is felt (it puts, as it were, the retentional trace into perspective, such that when a surprise arises from the unfulfillment of a protention, its presentification transforms the felt quality of the retained experience). Component processes of time consciousness thus qualify each other: they are inter-related in a “multiplicative” way (Gallagher and Zahavi, 2014). These processes operate synchronically and their interactive product manifests as a unified whole (Merleau-Ponty, 1945). It provides with a complex temporal field, a “specious” present in the thickness of which objects or events can be experienced with a time-extended quality (Varela, 1999a).

This threefold structure thus does not function as a mere sliding window (where protentions would become primal impressions which would further become retention). Retention, for instance, is not the intentional aiming of an absent phase of the outer object or event, for it is not possible to directly aim at something that is not actually there. Rather, retention refers to the just-elapsed phase of the experience of that object or event (Thompson, 2007). Because this experience had a threefold (primal impression – retention – protention) structure, what retention holds is a full threefold structure. Protention also has a threefold structure, for it intends what is anticipated to be about to qualify as retention, primal impression and protention. As component processes of the threefold (retention – primal impression – protention) structure “holds” the same threefold structure again (and so on), the dynamical flow of time-consciousness can be said to have a fractal structure (Gallagher and Zahavi, 2014). Fractality captures the self-similarity of a structure: constituting parts resemble the whole they form across multiple scales of observation or “zooms”. Vrobel (2011) also proposed a fractal interpretation of Husserlian accounts, in which “nows” (threefold structures) are nested into each other, and can be thought as different timescales or “levels of description”. Nesting nows provide nested nows with a (common) context in the light of which they are experienced. This multiscale structure is necessary for the current note of a melody to be meaningfully experienced not only in the narrow context of its predecessor, but also in the larger contexts of the melody or the whole piece it belongs to, or even the evening when it was listened to. In turn, nested nows can affect the experience of the contextual background in which they are embedded, such that the current note can modify how its embedding retentional background and its protentional horizon are experienced (especially if that note is surprising). Time as experienced thus does not follow a unidimensional, linear chronology: the temporal texture of lived experience thus has a multiscale, fractal topology.

Time consciousness has a multiplicative, self-referential structure: it makes references to its own retained pasts and anticipated futures. It is thereby a self-constituted flow: it manifests itself to itself, enabling the experience of the enduring quality of its own dynamics (the so-called “inner” time consciousness). This flow is therefore the “absolute,” irreducible, most fundamental level of time consciousness, and the necessary background out of which any experience can arise (Thompson, 2007). In other words, it is the pre-reflective structure of consciousness (Zahavi, 2003), the transparent background of our embodied perspective. This perspective is thus not just a point of view in the spatial domain: it is also a temporal perspective (Vrobel, 2011). The lived body thus has to be seen from the dynamical point of view of this flow. Because it presents itself as an affordance, the lived body is oriented toward what is anticipated to be about to be enacted. This orientation is underlain by the broken symmetry of time consciousness (to-be-fulfilled protentions intend what hasn’t been yet, in contrast to retentions that hold what has actually been). The dynamical structure of consciousness is thus always incomplete and moves forward, toward the complementarity of afforded anticipations. In this sense, time consciousness is enactive (Gallagher and Zahavi, 2014), pragmatically oriented toward (what) perception and action (could be). In turn, because perception and action emerge from this flow, they are imprinted by its dynamics and therefore have a similar structure.

Finally, because of the complex processes whereby components qualify each other dynamically, contents of experience affect its own intrinsic temporality (Gallagher and Zahavi, 2014). Indeed, think for instance about the fulfillment (or lack thereof) of a retained protention, and how it shapes primal impressions, their retentional background and their protented horizon. The flow of time consciousness thus makes present both the temporal content of experience and the temporal experience itself (i.e., both the “what” and the “how”). Outer and inner aspects of time consciousness thus co-constitute each other dynamically. Intrinsic temporality of experience thereby embodies the dynamics of the environment (Vrobel, 2011). Our dynamical perspective is thus relational as well.

Overall, embodiment constitutes an autonomous∼relational perspective whose dynamical background is self-referential, multiscale and multiplicative. This forms a pre-reflectively lived background from which we can inhabit the world. How does temporality manifest itself in the domain of the living? More specifically, how does the temporality of a complex organism emerge in a unified, coherent coordinated way? It is important to address this issue if we want to find out how time can be shared and what kind of temporality can be shared.

### TEMPORALITY OF THE LIVING

In this subsection, we discuss the processes that account for the features of the temporality of experiences, namely, its endogenous self-constitution, its non-linear, non-chronological unfolding, its multiscale, fractal nature, and its permeability to the environment’s temporality. We also introduce the dynamical concepts and models that will guide us toward a general understanding of how different temporalities can get coordinated and shared. We first refer to a simple, abstract model, in the light of which we discuss the temporality of both brain and behavioral dynamics.

In 1665, Huygens (Hugenii, 1673) deceptively observed that two pendulum clocks he designed for the sake of increased precision actually drifted apart when they were placed in isolated rooms. However, when they were placed on the same plank, their respective ticking converged until they reached synchrony, a state in which they then stayed. Though the clocks oscillated autonomously, they were flexible enough so as to be mutually affected by the vibrations they transmitted to each other (through the plank by which they were coupled). Because of the reciprocity of their interaction, clocks’ ticking became dependant on each other, and got attracted toward a common pattern. This pattern can then persist by efficaciously and commonly constraining clocks’ ticking. The stability of the collective system thus emerges from the interactions between its variable components. In dynamical systems terminology, such stability is captured by an “order parameter” (Haken, 1983) or a “collective variable” (Kelso, 1995), which measures the ordering of the relations among components. Emergent synchrony between coupled behaviors is actually ubiquitous in nature, though it manifests in obviously more complex ways (Pikovsky et al., 2001; Strogatz, 2003). Some of the most fundamental issues in brain and behavioral sciences are related to this phenomenon: how can large-scale coherent activity be formed in the brain out of its noisy basal functioning? How can coherent movements be performed despite of the numerous degrees of freedom they involve? The hypothesis according to which temporal coordination or “synergies” (Haken, 1983) emerge from the non-linear dynamics of interactions between coupled components (Kelso, 1995; Varela, 1995) has gained further and further support over the years. A brief look on brain and behavioral dynamics will help us to naturalize the temporality of lived experience as well as to understand how different components can coordinate in time by interacting.

As a result of non-linear interactions between neurons’ activity, brain oscillations can couple (Kelso, 1995; Varela, 1999a). Because brain signals are composed by a broad range of adjacent periodicities, oscillations whose frequencies are close enough can converge by reciprocally influencing each other (Buzsaki, 2006). This enables the emergence of large-scale synchronized patterns of activity, or assemblies. However, because of the detuning between intrinsic periodicities of neurons, coupling is weak: soft-assembled components quickly relax toward their intrinsic dynamics. Emergent assemblies are therefore transient, short-lived, and are followed by their own dismantlement (Buzsaki, 2006). This continuous reorganization is a signature of metastability, a regime characterized by the coexistence of contrasting tendencies: the integrative tendency of neurons to “cooperate” (i.e., to align their behavior through reciprocal interactions) and their segregative tendency to return to their intrinsic, autonomous functioning (Tognoli and Kelso, 2014). This allows for both the emergence of patterns of activity that are stable enough to be sustained over a significant period of time, and their flexible dismantling in order to make room for new patterns, which is important in the face of rapidly and ever-changing environmental conditions. Fluctuations thus enable the emergence of new stable (but flexible) patterns of coordinated activity: the variability of processes itself is therefore functional and adaptive.

The timescale at which large-scale assemblies are formed (hundreds of milliseconds) correlates well with the subjective impression of nowness: their short-lived maintenance allows for the thickness of the specious present (Varela, 1999a). According to Varela, the order parameter that captures the coherence of these soft-assemblies reflect an ordering that constrains future assemblies, a correlate of protention. The dynamic flow of brain activity is thus constrained and imprinted by the trace of ongoing and therefore previous patterns’ formations: it thereby constitutes retentional dynamics (Varela, 1999a). Varela (1999a; see also Freeman, 2000) designated other neurodynamical timescales: the micro-level of sensorimotor events (tenths of milliseconds) and the macro-level at which successive assemblies are coherently ordered (a few seconds). Interestingly, the many adjacent periodicities of brain signals exhibit a 1/f power law (the longer the periods of oscillations, the larger the amplitude of their contribution to the signal), a typical signature of fractal, metastable processes (Buzsaki, 2006; Werner, 2010). This encourages a view similar to Vrobel’s theory (2011) in which activities at different timescales are nested into each other, the slowest timescales of fluctuations constraining or enslaving the activity of the fastest ones (Penny et al., 2008). Overall, brain dynamics do not unfold according to a single timescale of operation. They evolve coherently, thanks to the interactions between fluctuating processes whose operations span multiple timescales. Brain dynamics thus seem to shape the felt envelope of time (Lutz et al., 2002) as well as to account for its complex multiscale texture.

How to achieve coherent behaviors despite of the numerous degrees of freedom they involve? Self-organization of component processes in a metastable regime would lead to “synergies” that are easier to guide (Bernstein, 1967; Haken, 1983). Bimanual rhythmic tasks support that hypothesis. Participants have been asked to give regular taps with both hands in alternance, by following the pace of a metronome (Kelso et al., 1981). When its frequency was increased until a certain critical threshold, patterns of movements suddenly shifted toward another organization: participants spontaneously, irremediably, and abruptly tapped with both hands in phase. The motor system bifurcated non-linearly from a bistable regime (two possible patterns of behavior coexist) to a monostable one (only one pattern can be stabilized in these circumstances). This metastable phenomenon can be modeled by the dynamics of a relational variable that measures the ordering of the relations among components’ activity (the relative phase between the limbs). Before the phase transition toward the uninstructed pattern occured, this relational variable started to fluctuate. This translates the loss of stability of the current pattern, which allows for a flexible reorganization of behavior (i.e., the sudden, emergent switching toward a more stable pattern). Behavioral dynamics thus seem to emerge from the self-organized interactions of components rather than from the sole properties of these components or from explicit central instructions (Kelso, 1995). In other words, and from a general point of view, common or coherent temporal patterns can emerge from the relational dynamics between various components: these collective patterns manifest themselves as attractors that dynamically co-ordinate in time components’ behaviors.

The multiscale, non-linear, fluctuating dynamics of brain and behavior are at odds with the classical view of time. Time is usually assimilated to its “objective” measurement and is subsequently described as a linear succession of isochronous units (Varela, 1999a). In the context of rhythmic behaviors, this view prompts to take stable, metronome-like regularity as the norm. Variability is thus seen as a deviation from that norm, as an error in cognitive measurements or motor implementations (Wing and Kristofferson, 1973; Delignières and Torre, 2009). While the tempo of music is indeed felt as having a stable quality in despite of the inherent variability of musicians’ performances (Large and Palmer, 2002), listeners experience these fluctuations as well, and not as errors or mere approximations. Rather, these fluctuations convey expressivity (Collier and Collier, 1996; Palmer, 1997; Iyer, 2002), a phenomenon also observed in mother-infant interactions (Gratier, 2003; Gratier and Apter-Danon, 2009). Variability of behavior thus makes sense. In fact, rather than being mere noise to ignore (whether statistically or cognitively), fluctuations of rhythmic performances exhibit a highly structured complexity. Studies on pianists (Rankin et al., 2009), drummers (Hennig et al., 2011, 2012) or non-musicians (e.g., Delignières et al., 2004; Lemoine et al., 2006) show that human tempo fluctuations are fractal: they display similar structures across scales of observation, with their amplitude decreasing with their frequency according to a 1/f law. The resulting rhythmic behavior is thus composed by the intertwinement of fluctuations of various amplitudes and periodicities, like waves enslaved in larger waves. Patterns of behavior are thus organized at multiple timescales, even when the task’s instructions target a unique timescale, such as the pulse.

The fractal structure of human temporality has been observed in many situations and seems to be the norm rather than the exception (for a review, see Van Orden et al., 2009). The hypothesis according to which fractal properties are generated by component processes (Pressing and Jolley-Rogers, 1997; Wagenmakers et al., 2004) is therefore fragile. Alternatively, fractality is thought to emerge from multiplicative interactions between processes that operate at multiple timescales (Van Orden et al., 2003; see Torre and Wagenmakers, 2009 and Delignières and Marmelat, 2012, 2013, for debates about these hypothesis). The latter hypothesis is supported by recent studies showing that behavioral dynamics actually exhibit multifractal properties (Ihlen and Vereijken, 2010; Dixon et al., 2012). While monofractal measurements only point out the co-presence of multiple timescales of fluctuations, multifractality captures the presence of contingencies across timescales of behavioral dynamics: underlying processes therefore interact at multiple timescales (Kelty-Stephen et al., 2013). Fractality has also been observed at multiple scales of organization and in many different measurements of the same behavior: this “pervasiveness” of fractality has been linked to metastability and the emergence of soft-assemblies (Kello et al., 2008; Kello and Van Orden, 2009; Holden et al., 2011). Indeed, while metastability reflects the balance of processes’ dependance and independance (their tendency to function in relation with each other versus autonomously), fractal fluctuations reflect the balance of temporal dependance and independance between processes through time and at different timescales. Fractality would thus be a signature of metastable dynamics. Relative dependance between processes and enslavement of local dynamics in fluctuations of larger timescales can create (long-term) correlations that fractal measurements capture. In contrast to uncorrelated fluctuations of independant processes, long-term correlations provide with a dynamical coherence that allows for a more robust unfolding of behavior. However, too rigidly correlated fluctuations (such as those introduced by strongly interdependent processes) wouldn’t let enough room for fast reorganization of behavior when demands of the environment change. Soft coupling of processes thus allows for a blend of stability and adaptive flexibility, and fractality illustrates optimal, healthy metastable dynamics whose complexity is often lost with pathology (Stergiou and Decker, 2011). The temporal baseline of biological dynamics is therefore complex, metastable and (multi-)fractal, rather than linear.

Brain and behavioral coordination thus doesn’t start “from scratch”: it doesn’t require the explicit control of all parameters or components involved in a specific pattern. Metastable dynamics provide with a background that “do something” for coordination. These spontaneous endogenous dynamics constitute a dynamical landscape that orients behaviors’ trajectories toward stable attractors (Kelso, 2009). In support of this view, it is this underlying dynamical landscape that is affected as a whole by learning (Kostrubiec et al., 2012). The role of intentional agency would thereby be to actively modulate this complex background of ongoing dynamics, in order to stabilize or destabilize its intrinsic tendencies (Kelso, 2002; see also Tschacher and Haken, 2007). This metastable background thus shapes what it is afforded to do and sense: it dynamically orients behaviors and experiences and is therefore a correlate of the lived body. Because it is constituted by processes that interact at multiple timescales and are nested into each other, metastable dynamics carry a portion of their own past in which they are embedded, and prefigurate a part of their upcoming trajectories. Metastable, fractal dynamics thus have a retentional-and-protentional structure that correlates well with the complex texture of the temporality of experiences (Vrobel, 2011). Our behaviors and the lived experiences they bring forth would be entangled in and shaped by these metastable dynamics. In turn, experiences and intentional agency can then act as global constraints that modulate and guide local endogenous dynamics (Thompson and Varela, 2001; Kelso, 2002). Living and lived embodiment thus co-constitute each other and form a dynamical embodiment whose temporality is complex, multiscale, (multi-)fractal, and retentional-protentional, rather than linear and chronological. This embodied temporality emerges as a whole from a complex but flexible background of relational dynamics, wherein processes interact with each other at multiple timescales.

### THE EMBODIED MIND IN THE TIME OF THE WORLD

So far, we considered the embodiment of time by subjects who were isolated from any environmental constraints (except the boundary conditions of experimental tasks). If embodiment is relationally constituted, its underlying dynamics should be imprinted by the environment’s temporality, as we show below.

Entraining to external temporalities happens very spontaneously at multiple timescales. For example, if we were isolated from the outside world, our wake/sleep cycles would not last 24 h (Czeisler et al., 1980). At a much smaller timescale, body movements can be unintentionally entrained to the oscillations of a moving room (that is merely displayed on a screen; Dijkstra et al., 1994) or even smaller stimuli (Lopresti-Goodman et al., 2007; Schmidt et al., 2007). Interestingly, synchronizing a limb in antiphase with a metronome whose frequency is increased brings forth the same dynamical features as tasks involving the synchronization of two limbs (Kelso, 1984). This isomorphism again suggests that patterns of coordination emerge from dynamics that exist at the level of the coupling (between limbs or between limb and metronome) rather than from the sole intrinsic properties of involved components.

Coordinating to the environment happens simultaneously and interactively at multiple timescales. For example, we synchronize in a more stable fashion to pulses that are embedded into larger patterns (Drake, 1993). Grouping pulses into larger patterns emerges spontaneously: participants do it during the performance of a mere pulse without any intention or awareness to do so (Parncutt, 1994) and perceive larger patterns that have no counterpart in objective information when they listen to isomorph, isochronous pulses (Bolton, 1894). Musicians’ expressive fluctuations reflect the organization of larger patterns as well (Repp, 1997) and enhance listeners’ coordination at these larger timescales (Drake et al., 2000). Synchronization to a pulse is also stabilized by the presence of subdivisions forming simple patterns (Repp, 2003) and destabilized when the fine-grained timing of these subdivisions is altered (Repp, 2008). More generally, the way one coordinates to a particular timescale of a stimulus reflects the temporal organization of that stimulus at other timescales (Large et al., 2002). We thus embody the stimulus’ temporality at these timescales as well, and this constrains the dynamics that operate at the targeted scale.

We do not just embody plurifrequential rhythms though (Toiviainen et al., 2010), but also the complex structure of their fluctuations. For instance, when participants synchronize to the tempo of a piece of music whose fluctuations are fractal, they produce taps whose variability quantifiably match that fractal structure. Conversely, participants’ taps do not exhibit a fractal structure at all in presence of a metronomic version of the same performance (Rankin et al., 2009). Participants’ taps also match the complexity of pulses of metronomes that fluctuate fractally (Hunt et al., 2014; Marmelat et al., 2014a) or chaotically (Stephen et al., 2008). Such a tight coupling is not the result of a mere “imitation” of the fluctuations by means of local adjustments. Rather, the multifractal structure of taps indicates that the pattern of coordination is more complex and emerges out of the interactions between processes operating at multiple timescales (Stephen and Dixon, 2011). Coupling with the environment thus seems to modulate the whole multiscale complexity of internal dynamics, even when the stimulation’s frequency is restricted to a narrow frequency band (e.g., a fluctuating pulse). As a result, multiscale patterns of coordination with the environment emerge as wholes. In this regard, (Large and Jones, 1999; Large, 2001, 2008; Large and Palmer, 2002) proposed models that account for perceptual and motor coordination to expressive fluctuations as well as to multiscale patterns. Endogenous dynamics are modeled by coupled autonomous oscillators whose respective intrinsic frequencies span multiple timescales. Their non-linear interactions enable the emergence of coordinated patterns of internal activity that span multiple timescales as well. The rhythmic signal acts as a sensory perturbation for ongoing internal activity. Coordination to that signal is thus modeled by the subsequent entrainment of internal oscillators to the periodicities of the signal. However, because oscillators are coupled with each other, the signal does not merely perturbate them individually, or frequency band by frequency band. Rather, the stimulus modulates the complex organization of endogenous dynamics as a whole, a general model whose essence captures the aforementioned empirical observations and fits our theoretical construction well.

On the one hand, multiscale patterns of coordination are constituted by an autonomous perspective: they emerge from the background of its *ongoing* endogenous dynamics (such that different patterns might emerge in the context of different ongoing internal dynamics, even when environmental circumstances are identical). On the other hand, patterns of coordination are constituted in relational dynamics: they are a product of the interactions with the world. Indeed, when sensory perturbations affect an agent’s internal dynamics, it modifies how these dynamics can later be modulated and what patterns can emerge out of it. This way, as in lived experience, inner and outer temporal dynamics co-constitute each other irreducibly. Endogenous and relational dynamics are thus intertwined such that patterns of coordination are both autonomous and relational. Because they are constrained by the dynamical traces of what is going on endogenously and thereby by the traces of agent∼ world relational dynamics, patterns of coordination are retentional. Internal dynamics thus embody the regularities of the environment in its own fluctuating activity. Because sensory perturbations are experienced in the light of this ongoing activity, this dynamical backgound provides with implicit anticipations, or protentions. For instance, when internal dynamics are modulated and stabilized by a certain pattern of perturbations that is repeated, a sudden difference in the stimulus introduces a difference in the agent∼world’s relation: it unfulfills the protention embodied in the agent’s ongoing internal dynamics. Dynamical embodiment of external temporalities thus allows for a strong, multiscale coordination with the environment (Dubois, 2003; Stepp and Turvey, 2010). Dynamical models that blend internal and relational dynamics therefore provide with a framework for both perceptual and motor coordination to the world. In this regard, the relations between participants’ patterns of activity and patterns of stimulation were investigated [e.g., the relation between patterns of response times and the temporal patterning of successive stimuli (Holden et al., 2011) or the relative phase between participants’ taps and the metronome they follow (e.g., Chen et al., 2001)]. In these cases, the dynamics of these relations exhibit fractal fluctuations as well, in a way that strongly depends on the temporality of the context of the task (Holden et al., 2011). This further points out that soft-assembled, metastable patterns of coordination emerge at the level of the whole agent∼world coupling.

Overall, interactions between processes operating at multiple timescales form an endogenous background of metastable dynamics. It is from this background that temporal coordination of activity and experiences can emerge. It is therefore the background of our autonomous perspective: it orients the dynamics of our embodiment (i.e., both experiences and behaviors). Because it is modulated by the dynamics of its relations with the world, this “dynamical landscape” embodies the environment. Relational dynamics thus shape the dynamical landscape of our “sensorimotor habitat” (Buhrmann et al., 2013). The coordinated inhabitance of the world we enact is therefore autonomous∼relational. Embodiment is thus a dynamical phenomenon, and it is the temporality of the behaviors and the experiences it gives rise to that can be shared in human interactions (i.e., it is in the course of these dynamics that we can be together). To address this issue in more depth, we first discuss how embodiment and intersubjectivity relate to each other. We then question the temporality that emerges from the dynamics of their relation, and how this temporality is embodied by interacting subjects.

## EMBODIMENT OF INTERSUBJECTIVE TIME

### EMBODIMENT AND INTERSUBJECTIVITY

When we meet an other person, “what” we interact with is a “who” (McGann and De Jaegher, 2009): another embodied perspective. This transforms the dynamics of our embodiment in two contrasting but complementary ways. On the one hand, because the sensory-motor affordances of our respective embodiments are similar, we are subtely sensitive to each other’s behaviors and to a similar world. On the other hand, our very embodiment makes alterity persist indefinitely: our respective embodied perspectives always differ (especially when they aim at one another). In this subsection, we detail the phenomenological implications of these two aspects successively, and then present experiments that track their underlying dynamics.

During our mutual encounters, part of my transparently lived body (e.g., my looking eyes, my expressing face) becomes a visible living body for the other (Lenay, 2010, who we closely follow in the next two paragraphs). Because the other is sensitive to my activity, the expression of my lived experience through my visible living body affects him and thereby changes his own lived experience. I can thus modulate and participate to the other’s experience. The expression of his own experience is visible to me as well (especially the expression of the *changes* I induced in his experience). I am therefore also living experiences to which the other participates, in a way to which I participated to upstream. The other thus becomes part of my embodied coupling with the world: I do something to him that changes something for me. This way, I can pragmatically experience the other, I can enact him (I bring forth an experience of the other that emerges from the consequences of my activity toward him). By the reciprocity of this pragmatic link, we become part of each other’s embodied coupling: our respective embodiments become dynamically contingent of each other (we dynamically co-determine each other’s behaviors and experiences). When we interact, we thus mutually enact each other (Varela, 1999b; Thompson, 2001), so that we can participate to and mutually incorporate each other’s embodied perspective (Merleau-Ponty, 1945; Fuchs and De Jaegher, 2009). It is thus by interacting that we can share experiences, activities, meaning, and so to speak, points of view (De Jaegher and Di Paolo, 2007).

Whatever I do changes the other: he thus constantly escapes my intentions toward him (Lenay, 2010). In return, changing the other also affects me. During our interactions, I thus change myself as well, so that any of my intentions glides in the interaction process itself, wherein they get remolded. By interacting, I therefore also escape myself (hence the difficulty of applying a prepared plan of conversation once the actual encounter is unfolding). Our experiences of each other and ourselves are thus always broken, incomplete and escape us so that our interactions keep moving forward. Because the visible effects we have on each other are transparently caused (by our pre-reflectively lived body), part of the very linkage of our respective embodiments escapes both of us as well. The dynamics of our relations thereby acquire an autonomy of their own (De Jaegher and Di Paolo, 2007). Because these relational dynamics affect us simultaneously, they can efficiently coordinate our respective embodiments and constitute our behaviors and experiences in a common fashion, from a common dynamical background (De Jaegher et al., 2010). Our dynamical embodiment is thus shaped by the dynamics of our relation: we embody collective dynamics. In this sense, not only do we incorporate each other’s perspective, but we also transparently incorporate the dynamics of the interaction process itself (De Jaegher, 2009). In other words, the dynamical background of our embodied perspective is constituted in the process of interaction. The pre-reflectively lived landscape that orients us in our sensorimotor habitat is therefore interactively shaped (Kyselo and Tschacher, 2014).

Our respective embodiments thus become contingent of each other not only because of their congruence, but also because of their broken symmetry. On the one hand, incompleteness of relational dynamics keeps the interaction moving forward. The resulting dynamical autonomy of the interaction process can thereby “bonds” our respective embodiments. On the other hand, this incompleteness makes alterity persists. The interaction process thus always involves us personally and still imply our autonomous agency (De Jaegher and Di Paolo, 2012). While embodiment is constituted in and by relational dynamics, it is at the same time these very relational dynamics that have to be actively regulated. As it depends on the other and its the complementary involvement in the process of interaction, the active modulation of interpersonal coupling escapes us. As an individual effort, it is always incomplete. It is a co-regulation of an irreducibly collective process. The co-regulation of our coupling entails a dynamical congruence such that an even more fine-grained sharing of embodied dynamics becomes possible. Further, because the process to regulate is collective, sharing its modulation has a quality that is proper to the interpersonal domain: it makes sense in itself. Sharing experiences, activities or meanings is thus not just about the content. It involves an inter-enactive process whose dynamics have a proper quality that makes sense on its own. Because its underlying dynamics participate to our embodiment, and because we can experience the consequences of the co-regulation of these dynamics, this intersubjective quality can also make sense to us personally.

**FIGURE 1:**
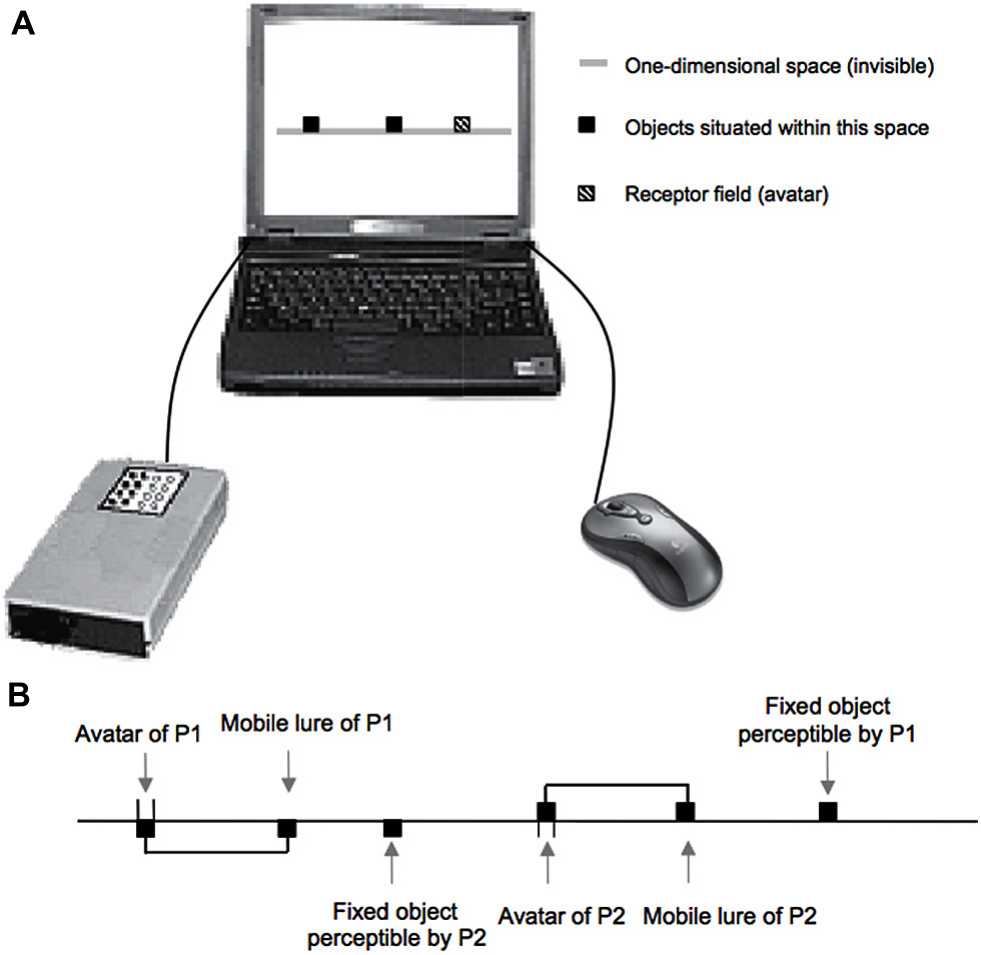
**Experimental set-up of Auvray et al. (2009). (A)** Technological device set up by Auvray et al. (2009). **(B)** Schematic illustration of the (invisible) virtual environment. Movements of the mouse displaced the receptor field of an avatar in a one-dimensional virtual environment. Whenever the receptor field overlaps the current position of another entity, the participant that manipulates this avatar receives a tactile stimulation. Reprinted from Auvray et al. (2009), with permission from Elsevier.

Auvray et al. (2009) empirically tracked the general dynamical structure of human interactions. Pairs of blindfolded participants manipulated a device that reduced their sensorimotor coupling to a strict minimum: each participant moved a mouse that displaced an avatar in a virtual environment and participants received a unique type of tactile stimulation whenever the receptor field of their avatar overlapped the position of an entity in that virtual environment (**Figure 1**). There was thus only one bit of information (0: no stimulation; 1: stimulation). In this context, participants couldn’t distinguish if the stimulations they received resulted from the crossing of their partner, or from the crossing of a lure that imitated the partner’s displacements. However, participants met each other a lot more often than they met the lure: they found each other without knowing they did. The difference between the two situations emerges at the collective level. The lure is disembodied: it doesn’t receive any stimulations that modify the internal dynamics of its behavior. Conversely, the partner is embodied and the overlap with its receptor field leads to a mutual stimulation. Even if all participants participant ignore what they do for the other (Lenay, 2010), they affect each other’s behaviors. They thereby got attracted toward a common pattern of behavior (a reversal of movements around the source of stimulation). In other words, they were oriented and coordinated by the mutual and common effects of the interaction process, without any awareness of the dynamical situation in which their behavior got entangled. This illustrates how the incompleteness of the encounter (i.e., what I do for the other escapes me, as well as what the dynamics of our patterns of relations do for us) allows for the interaction to move forward *on its own*. The coordination of behavior that is observed externally can thus emerge from the process of interaction and/or its regulation (Froese et al., 2012; Lenay and Stewart, 2012; see Auvray and Rohde, 2012, for a review of replications of the above experiment with both human participants and artificial agents). Boker et al. (2009) captured such kind of phenomenon in a somewhat more ecological experiment. They reduced the visible expressivity of one of two conversational partners by resynthesizing the movements of its realistic avatar. This effect was transparent to him, but visible to his partner, who enhanced the amplitude of his own movements, as if he were compensating for this lack of expressivity. The complementary regulation of coupling dynamics then became explicit as both partners ended up enhancing the expressivity of their movements, without any awareness to do so. Their behaviors became thus entangled in relational dynamics between their embodiment in a way that escaped them.

Relational dynamics can attract agents’ internal dynamics toward behavioral regions that aren’t reachable or attracting outside of a mutually engaging situation (Froese and Fuchs, 2012; see Laroche and Kaddouch, 2014, in the domain of musical pedagogy). The process of interaction can thus transform individual repertoires of behaviors by shaping the underlying dynamical landscape that orients them. Relational dynamics thus modulate our affordances such that we embody collective dynamics (i.e., collective dynamics are part of our embodied coupling). In the experiment of Auvray et al. (2009) though, the embodiment of collective dynamics didn’t seem to entail a distinct experience (participants didn’t distinguish the lure from the partner). With more precise measurements of lived experience and by explicitly encouraging participants to collaborate, Froese et al. (2014a) observed that they could discriminate each other from the lures. Partners relied on the dynamical complementarity afforded by their interaction and actively co-regulated their coupling. Judgments were thus based on the enactive experience of irreducibly collective dynamics. In support of that interpretation, mutual recognition increased the clarity of experience of the other’s presence: collective patterns modulate personal experiences. Subjects thus embodied relational dynamics in the full sense of the term: their behavior was livingly oriented by the interaction process, and they had a distinct experience of the relational dynamics they co-regulated and in which they were caught.

If, by interacting, we can participate to each other’s embodiment, then we participate to each other’s pre-reflective dynamical background. The temporalities of our respective embodiments should thus get coordinated as an effect of interacting. Because the process of interaction escapes us, it can bring forth a temporality of its own: a properly intersubjective time (Gratier and Apter-Danon, 2009) that emerges from interpersonal relational dynamics. Because the process of interaction coordinates us, we can also embody this temporality (it participates to our dynamical background). By actively regulating relational dynamics that affect us, we can experience this intersubjective temporality by ourselves. It is precisely because this regulation partly escapes us and involves the complementarity of our respective activities that we can experience its intersubjective quality. This is in the course of such an intersubjective time that we can be together. This intersubjective quality has to be brought forth before it can be experienced and thus shared in a dynamical and embodied way. Being together (as experienced enactively) can therefore be hypothesized to be the experience of the coordination of our dynamical embodied perspectives that emerges from our relational dynamics and their co-regulation. More precisely, in light of the previous sections, intersubjective time should yield autonomous∼relational patterns of coordination underlain by multiscale metastable dynamics. In the next subsection, we discuss empirical results that support this hypothesis.

### EMBODIMENT OF INTERSUBJECTIVE TIME

In this subsection, we address the issue of the embodiment of intersubjective time. We briefly review the empirical literature that supports hypotheses that emerged from the framework that has been built so far. We first point out that behavioral dynamics coordinate during interpersonal interactions, so that it leads to the emergence of a common, shared temporality of behavior. Afterwards, we verify that this coordination emerges from the metastable relational dynamics of between-persons interactions. Next mutuality of interaction is shown to play a proper role in these dynamics. This leads us to point out that the experience of the intersubjective dimension of interpersonal timing is enacted thanks to the co-regulation of the interaction process. It therefore requires the personal but flexible engagement of individuals. We then discuss the functional role of fluctuations in interpersonal coordination dynamics. Finally we show that these dynamics and their co-regulation coordinate interacting persons in a multiscale and multiplicative way, and that this forms a shared dynamical background in which behaviors and experiences are entangled.

The temporal coordination of individual behaviors manifests spontaneously in our daily interactions (Condon and Ogston, 1966), most often in a rhythmic way (Condon, 1986; Bernieri and Rosenthal, 1991; Gill, 2012). For instance, both newborns and adults tend to synchronize their movements to the speech of their interlocutor (Condon and Ogston, 1971; Condon and Sander, 1974). Behavioral coordination is multimodal (Kendon, 1970; Barbosa et al., 2012; Louwerse et al., 2012; Bangerter and Mayor, 2013) as well as physiological (Guastello et al., 2006; Feldman, 2007; Feldman et al., 2011; Müller and Lindenberger, 2011). A tight temporal coupling is even observed in breathings during turn-taking (McFarland, 2001) and speech rates converge (Street, 1984). Whereas conversations seem to be structured by an alternance of roles (speaker vs listener), behaviors are thus underlain by a common temporal framework. Relational dynamics seem to attract individual temporalities toward a shared timing (Deschamps et al., 2012; Froese et al., 2012). In laboratory settings, individually prefered tempi indeed tend to move toward a common ground even when people coordinate unintentionally and without awareness to do so (Oullier et al., 2008).

If coordinating in time isn’t the proper aim of daily interactions, how does it arise? In the light of the previous sections, we would expect that temporal coordination of behaviors emerges spontaneously from the self-organization of between-persons relational dynamics. This hypothesis is supported by numerous studies (for reviews, see Oullier and Kelso, 2009; Delaherche et al., 2012; Schmidt et al., 2012; Dale et al., 2013; Lagarde, 2013). For instance, when pairs of participants oscillate their legs in anti-phase (opposite directions) at an increasing frequency, their coupling becomes unstable near to a critical threshold; phase wandering between attractors or abrupt transitions toward more stable patterns is observed (Schmidt et al., 1990), a typical signature of self-organized dynamical systems that are modeled by non-linearly coupled oscillators (see also Schmidt and Turvey, 1994; Amazeen et al., 1995). Interpersonal patterns of coordination thus follow the same dynamical laws than bimanual patterns or unimanual-metronome patterns (see also Mottet et al., 2001; Black et al., 2007; Richardson et al., 2007a). This isomorphism suggests again that coordination emerges from the dynamics of interaction rather than from the specific properties of the coordinated components. Such synergistic effects have also been observed in more ecological tasks such as martial arts and hand clapping games (Riley et al., 2011), rocking chairs (Richardson et al., 2007b; Frank and Richardson, 2010), in language games that imply turn-taking (Schmidt et al., 2011) or in problem-solving tasks (Shockley et al., 2003; Richardson et al., 2005; Coey et al., 2011; see also Richardson et al., 2008; Shockley et al., 2009; Fusaroli et al., 2014). During sport activities, whether players are opponents or not, the dynamics of their coupling spontaneously self-organize and attractors emerge from their collective dynamics as well (Bourbousson et al., 2008, 2010a,b; Travassos et al., 2011; Yokoyama and Yamamoto, 2011; Okumura et al., 2012; Duarte et al., 2013; García et al., 2013). Whether intended or not, interpersonal coordination is thus underlain by a similar dynamical landscape constituted by attractors of collective dynamics (Schmidt and O’Brien, 1997; Richardson et al., 2007b; Oullier et al., 2008). The spontaneity of interpersonal dynamics is such that coordination also emerges when participants are specifically instructed not to do the same movements as their partner (Boker and Rotondo, 2002; Issartel et al., 2007). Movements unintentionally coordinate even when participants attend to a different external pacer, up to the point that the very reorganization of their own behavior tended to occur through simultaneous phase transitions (Varlet et al., 2011). The coordinative efficacy of the process of interaction is thus difficult to escape from. Because it happens most often without any awareness on our behalf, it precedes its explicit experience and thereby its regulation. Individual behaviors thereby seem to be entangled in the relational dynamics of their coupling. Intention and attention might then guide the regulation of this metastable background of collective dynamics in order to stabilize it (see Temprado and Laurent, 2004).

Relational dynamics of interpersonal interactions involves two autonomous embodied perspectives and are thus bidirectional. Studies on interpersonal coordination dynamics rarely took this aspect into account: usually, the comparison is made between coupled and non-coupled situations. The enactive approach emphasized the role of the very mutuality of interactions as a source of coordination (e.g., Froese and Di Paolo, 2008), which points out the properly interpersonal dimension of this phenomenon. Murray and Trevarthen (1985) and Nadel et al. (1999) evidenced the importance of the mutuality of the interaction process. Infants and their mother interacted through a TV-monitor, until the live retransmission of the mother’s behavior was replaced by a recording of her behavior made during the same interactional sequence. Though infants observed the exact same behavior of their mother in both situations, they reacted very differently when they faced the recording, displaying anger and frustration. Probably because they could not experience their own contribution in the regulation of the relational dynamics, they lost interest in interacting with their non-responsive (recorded) mother. This happens even when her image is delayed by three seconds only (Henning and Striano, 2011). In adult video-conferences, slight delays in the transmission of information can destabilize interpersonal coupling dramatically too (Nijholt et al., 2008). The mutual, simultaneous sharing of the interaction process is thus critical to interpersonal coordination, which can therefore not be reduced to purely individual processes.

Collier and Burch (1998) made the general prediction that bidirectional interactions between complex systems should yield “more effects for less effort” (i.e., enhanced coordination for less energy dissipation) than unidirectional interactions where only one system can be affected by the other. Indeed, mutual interactions entail more accurate and/or stable coordination than unidirectional ones (Cummins, 2009; Konvalinka et al., 2010; Shikanai and Hachimura, 2012; Hart et al., 2014), or than interactions where participants had to follow a partner who has a metronomic cue in his headphones (Oullier et al., 2003). Moreover, when mutual interactions are compared to unidirectional ones, increased stability of coordination at the level of the interpersonal coupling is accompanied by decreased fluctuations at the individual level (Hart et al., 2014), confirming the general “more effects for less effort” hypothesis (Collier and Burch, 1998). It seems that relational dynamics enable the (potentially or partly self-organized) co-regulation of each other’s variability, as if it was the coupled system’s whole variability. Our influence on the other, his responsiveness and the relational dynamics it entails thus do something for our coordination: it lays a background of collective dynamics that orient our inter-actions. By interacting, we co-regulate this metastable background, and thereby co-organize the dynamics of each other’s embodied background. This permits to unload part of the coordinative process on the dynamics of interactions themselves. Our embodiment is thus such that it can benefit from the (self-organized and co-regulated) complementary dynamics of each other’s actions. Conversely, unidirectional coupling rigidifies the situation. In this situation, variability cannot be organized collectively: the entire inflexible variability of the unresponsive partner has to be accomodated by the other on top of his own fluctuations. As already stated, stability (at the collective level) thus involves flexibility (at the individual level).

Unilateral and mutual embodied coupling thus have distinct phenomenologies. However, during concrete interactions, these two typical situations are extremities of a whole “spectrum of participation” (Di Paolo and De Jaegher, 2012). Different degrees of involvement can indeed be invested in the regulation of the interaction process. Interacting therefore implies participating to the modulation of the interaction process by modulating our participation to that process. Attention could thus be directed toward different aspects of autonomous∼relational patterns of coordination. Indeed, leaders (or socially dominant personalities) seem more focused on their own behavioral temporality: they display less fluctuations and thereby interact in a more rigid fashion than “followers” (Schmidt et al., 1994; Fairhurst et al., 2014; see also Sacheli et al., 2013). Followers pay more attention to the stability of the interaction process itself (Fairhurst et al., 2014). However, participants classified as “socially dominated” can be overresponsive (by taking the interaction process too much in charge; Schmidt et al., 1994). This might not leave enough room for the personal involvment of the other in the co-regulation of relational dynamics and the variability of behaviors that underlies it (Repp and Keller, 2008). For instance, social anxiety disorders entail difficulties in intentionally leading a coordination task (Varlet et al., 2014).

The coordinated regulation of interactions thus implies moderate contingencies, that is, flexible deviations from strict synchrony (Gratier and Apter-Danon, 2009). Such flexibility of the interaction process is also observed in mother-infants interactions, where moderate contingencies are both preferred and preferable for communication and development (Jaffe et al., 2001; Gratier, 2003; Hane et al., 2003; Gratier and Apter-Danon, 2009). Interpersonal rhythmic structures facilitate and guide coordination by providing embodied coupling with anticipatory dynamics. The emergence of interpersonal rhythms thus allows for dynamical backgrounds of embodiment to converge and to be organized with congruent retentions and protentions. Flexible fluctuations are functionnal too. They provide with surprises and make the interaction process incomplete (protentions are not entirely fulfilled). This incompleteness then requires the active engagement of participating individuals in the co-regulation of their relational dynamics (Deckers et al., 2012). Further, flexibility also permits to repair coordination breakdowns by reorganizing the interaction process. Optimal relational dynamics are thus a balance of stability and flexibility, a compromise between random fluctuations and strictly metronomical rhythms. In other words, interpersonal relational dynamics are metastable. This regime of interpersonal coordination leaves enough room for autonomy, such that subjects can experience their interactions in the background of their own dynamical embodiment. It also leaves enough room for relational dynamics to bring forth a temporality of their own. The co-regulation of these dynamics provides with a common dynamical background that modulates and coordinates autonomous embodiments. In this regard, spontaneous imitations of each other’s behavior entail the temporal coordination of brain dynamics themselves (Dumas et al., 2010; for reviews of inter-brain synchronization studies, see Dumas et al., 2011 and Konvalinka and Roepstorff, 2012). Autonomous and relational dynamics thus co-constitute each other, such that, by interacting, we co-enact a time whose sharing can be experienced inter-actively.

If the interaction process entails metastable relational dynamics, the latter should exhibit multiscale multiplicative dynamics. The presence of coordination of multiple behavioral cyclicities has indeed been observed during conversational interactions (Newtson, 1993; Sadler et al., 2009). Moreover, relational dynamics observed in movements had significant interpersonal meanings such as dominance and affiliation (Sadler et al., 2011). Mother-infants interactions are also coordinated at multiple timescales (Malloch, 1999; Gratier, 2008; Gratier and Apter-Danon, 2009): they follow an implicit pulse, and form broader phrases as well as longer narrative cycles of vocal and behavioral exchanges. Interestingly, the behavioral timescale of micro-expressivity, pulses and phrases correlate well with the neurodynamical scales described by Varela (1999a). Further, dynamics at work at these behavioral timescales seem to interact with each other. For instance, the lack of expressivity of deviations from isochrony at the pulse level has long-term effects on the overall quality of coordination (Gratier and Apter-Danon, 2009). The perturbation of the precise simultaneity of time has deleterious effects on the overall temporal organization of adult interactions, including turn-takings (Ruhleder and Jordan, 2001). On top of being multiscale, the interaction process thus exhibit signs of multiplicative dynamics. Indeed, in interpersonal motor tasks, relational variables such as relative phase or cross-correlation of periodicities of behaviors exhibit fractal structures (Hennig, 2014). Further, Ashenfelter et al. (2009) observed that head movements of conversational partners have a multifractal structure. It consisted in two fractal scalings: one at the level of local dynamics (short timescales) and the other at a more macro level. Ashenfelter and colleagues interpret this result as an indication of the presence of both coordinative processes and role alternance (or symmetry formation and symmetry breaking). The dynamical background that underlies interpersonal interactions is thus metastable: it is characterized by a dynamical blend of stable integration and flexible segregation of individual behaviors (Kelso and Engstrom, 2006).

If we participate interactively to each other’s dynamical embodiment, then the whole complexity of our dynamically embodied perspectives should get coordinated. In general, interacting complex systems are expected to match the very complexity of each other’s dynamical organization (West et al., 2008). Indeed, a flexibly fluctuating and responsive metronome (built on non-linearly coupled oscillators) can reinstate fractal dynamics of Parkinson diseased patients’ gait at a normal level, whereas this “healthy” complexity is lost as a consequence of this pathology, as evidenced in absence of a metronome or in presence of an unresponsive one (Hove et al., 2012). Mutually coupled participants match each other’s fractal dynamics of behavioral fluctuations as well (Marmelat and Delignières, 2012). Participants also match the fractal dynamics of their partner when they are unidirectionnally coupled (Marmelat et al., 2014b), but to a far lesser extent than mutually coupled participants (Laroche, unpublished). Co-regulated relational dynamics thus entail an attraction of complex internal dynamics toward congruent patterns of coordination. Dynamically and actively shared patterns of coordination that are both autonomous and relational thus emerge as wholes.

Overall, the complex temporalities that underlie our behaviors can be strongly coordinated at multiple interacting timescales. As a consequence, the backgrounds of our respective embodiments are dynamically bonded in a very subtle way. It is as if we were mutually attracted toward a common manner of “inhabiting” and shaping the time in the course of which we live. This could be hardly explained by individual capacities that would seek to mimick such complex dynamical structures. This phenomenon rather seems to emerge from relational dynamics between dynamical embodiments whose respective complexities converge by attraction and co-regulation. As even chaotic signals can synchronize their complex behavior (Strogatz, 2003), this is eventually not a surprising phenomenon.

If complex behavioral dynamics influence each other and are attracted toward collective patterns, their retentional and protentional structures should mutually orient and shape each other, and thereby be enactively shared. The pre-reflective dynamical background of experience should thus be shaped by the interaction process (Obhi and Hall, 2011). Interpersonal coordination dynamics are indeed experienced meaningfully (Gratier and Apter-Danon, 2009; Gratier and Magnier, 2012). Their co-regulation can lead to a coordination of personal experiences (Markey et al., 2010; Wiese et al., 2010) as well as to experiences of interpersonal connection (Hove and Risen, 2009; Marsh et al., 2009; Miles et al., 2009; Paladino et al., 2010; Ramseyer and Tschacher, 2011; Watanabe et al., 2011; Vacharkulksemsuk and Fredrickson, 2012). In turn, the embodiment of collective dynamics favor cooperative and pro-social behaviors (Wiltermuth and Heath, 2009; Kokal et al., 2011; Valdesolo and Desteno, 2011; Behrends et al., 2012). Unfortunately, precise first-personal descriptions of the lived experience of being together in time still lacks (but see Froese et al., 2014b). However, it is precisely because relational dynamics participate to each other’s experience that the interaction process can be appropriated and co-regulated (Laroche and Kaddouch, 2014; Froese et al., 2014a). Being toghether in time is thus inter-enacted: by interacting, we embody collective dynamics that coordinate our behaviors and experiences, and we participate actively to the regulation of that process. By co-regulating our embodied relational dynamics, we can co-enact a shared world of significance in which to be together. With this final remark in mind, let us now summarize and conclude this paper.

## CONCLUSIVE DISCUSSION

In this paper, we proposed a dynamical and embodied, enactive framework for the understanding and the investigation of the phenomenon of being toghether in time. We first defined embodiment as being both a living and a lived phenomenon that emerges from agent∼world coupling. Embodiment provides us with a perspective on our relations, a pre-reflective dynamical background on the basis of which we can enact the world through autonomous embodied interactions. This background is constituted by the self-organization of component processes whose interactions span multiple timescales. From the point of view of the living, temporality has a shape that is thus totally different from the “physical time” (Bailly and Longo, 2008; Holden, 2013). As a result of an underlying metastable regime, the temporality of the living is multiscale, multiplicative, (multi-)fractal. Behaviors and experiences thus carry the imprint of these complex dynamics in which they are entangled. This dynamical background is at the same time co-constituted by the dynamics of our relations with the world. Whole autonomous∼relational patterns of coordination thereby emerge, so that inner (“subjective”) and outer (“objective”) temporalities co-constitute each other dynamically.

During between-persons interactions, relational dynamics can self-organize and escape us. This gives rise to attractors of behavior in the shared dynamical landscape that we enact and navigate or inhabit together. By exerting a mutual attraction on their underlying temporalities and by coordinating them in time, relational dynamics can constitute individual behaviors and experiences. In short, by interacting, we embody collective dynamics. Mutuality of interaction further allows for the co-regulation of each other’s background of variability, as well as the emergence of a time that is properly intersubjective. The very complexity of our dynamical embodiments can thereby be inter-enactively shaped and thereby shared. This enables a strong coordination that is not a mere local synchrony (it is not a succession of synchronous states), but is extended in time at multiple interwoven scales. Since intrinsic dynamics of temporal experiences and the content of these experiences co-constitute each other, by interacting we can participate to each other’s pre-reflective dynamical flow. In other words, thanks to the inter-enactive process, retentions, protentions and their multiplicative interplay can be actively and dynamically shared (not in the sense that we have an informational duplicate of each other’s dynamical flow, for such a flow always emerges from its own background, but rather in the sense that we mutually shape each other’s pre-reflective dynamical background). Part of our experiences are therefore embodied in each other’s retentions and protentions. A co-enacted dynamical landscape thus emerges and forms a background of collective dynamics that brings forth a properly intersubjective time and coordinates its personal embodiment. Behaviors and experiences are thus entangled in this collective metastable background. By actively co-regulating these relational dynamics and by experiencing the effects of this co-regulation, we can experience the intersubjective dimension of this shared time as well as experience this sharing.

Overall, being together is neither a mere co-presence in the physical space, nor a mere temporal correlation of activities in the physical time that can be observed from an external point of view. It is the co-regulated and skillful inhabitance of the complex, metastable dynamical landscape that emerges spontaneously from the meeting of our embodied perspectives. Being together has thus to be enacted, that is, it has to be actively, dynamically and autonomously but relationally brought forth. In short, we can only experience being together through our inter-enactive engagement. In turn, this experience carries the imprint of the collective dynamics that emerge from this inter-enactivity. However, precise phenomenological descriptions of being toghether in time still lack. The recourse to more fine-grained phenomenological methods (e.g., Petitmengin, 2001) could guide fruitful empirical and modeling researches. Indeed, it is yet not clear how the temporal complexity of behaviors as measured gives rise to, is influenced by, or at least is correlated with clear and meaningful felt qualities (but see Lutz et al., 2002, in the intrapersonal domain).

Complex multiscale dynamics of interpersonal interactions have not been much addressed yet. Notwithstanding, it is a promising avenue of research. For instance, deficits in social coordination might be rooted in a loss of complexity, possibly at both the individual and the collective level (for recent dynamical studies, see Lazerges et al., 2011; Varlet et al., 2012, 2014; Lavelle et al., 2013; Marsh et al., 2013). If we take the interaction process seriously, as well as the complexity that underlies our dynamical embodiment, treatments of cognitive disorders might be improved. For example, rhythmic auditory stimulations improve the linguistic performances of children diagnosed with developmental language disorders (Przybylski et al., 2013). Further, fractal metrics can distinguish between dyslexic and normal readers in a word-naming task (Wijnants et al., 2012). Couldn’t a flexibly fluctuating and responsive rhythmic device improve performances even more, in the vein of the aforementioned work of Hove et al. (2012) with Parkinson Disease patients? If relational dynamics coordinate individual behaviors by modulating their underlying endogenous dynamics, responsive devices might entail more healthy dynamics, whereas part of the burden of coordinating to this device could be unloaded onto the interaction process itself.

Finally, coordinating in time leaves traces on embodied dynamics after the interaction itself (Oullier et al., 2008; Hove et al., 2012) on top of explicit traces of the partner himself (Macrae et al., 2008; Miles et al., 2010). Recurrent interactions and the temporal coordination they entail might enable the stabilization of interactional repertoires as well as the emergence of long-term and large-scale bonding such as those found in cultural practices and habits (Gratier and Apter-Danon, 2009; Gratier and Magnier, 2012). Dynamical models of embodied interactions thus might also play a significant role in the understanding of socio-cultural phenomena that are observable at larger timescales (Aguilera et al., 2013; Cao et al., 2013).

## Conflict of Interest Statement

The authors declare that the research was conducted in the absence of any commercial or financial relationships that could be construed as a potential conflict of interest.

## Footnotes

1 The tilde sign used in this text is a reference to Kelso and Engstrom (2006). It denotes that paired concepts are dynamically related to each other: the separate understanding of each concept remains incomplete as long as its complementary aspect is not taken into account
